# Understanding intention and use of digital elements in higher education teaching

**DOI:** 10.1007/s10639-023-11798-2

**Published:** 2023-04-29

**Authors:** Wieland Müller, Michael Leyer

**Affiliations:** 1 Institute of Business Administration, University of Rostock, Rostock, Germany; 2 Digitalisation and Process Management, University of Marburg, Marburg, Germany; 3 School of Management, Queensland University of Technology, Brisbane, Australia

**Keywords:** Digital learning, Higher education, Reasoned action approach, Intention beliefs, Usage effort

## Abstract

Digital elements are being increasingly used in higher education teaching, but the intention and their actual use vary depending on the lecturers. We used the reasoned action approach to understand the beliefs and intentions behind the use of digital elements in this context. We conducted a quantitative survey in which university lecturers shared their intention concerning the use of digital learning elements and indicated their actual use. The results confirm the influence of attitude, perceived norms, and perceived behavioral control on the intention to use digital learning elements. However, we also identified an intention–behavior gap: Only one-time effort to become familiar with digital elements has a significant impact on actual usage. We conclude that, above all, teachers must first be given the opportunity to become familiar with digital learning elements to be able to use them effectively. Understanding why such an intention–behavior gap exists should be the aim of future studies.

## Introduction

Due to ongoing digitalization, new possibilities have emerged for the delivery of education. Digitalization of education can provide students with more efficient and convenient ways to achieve their learning goals (Dumford & Miller, 2018). In the past decade, digital applications have found their way into classrooms and lecture halls, where they are being increasingly used (Rafiq & Ameen, 2012). Although various digital solutions have been available for many years, they are only partially used in teaching (Eickelmann & Vennemann, 2017; Zhang, 2020). The COVID-19 pandemic has largely contributed to the increase in digital teaching that has taken place in recent years (Bond et al., 2021). However, it has also raised some questions concerning how digital teaching will continue to develop in the future (Radhamani et al., 2021). It can be assumed that, when the COVID-19 pandemic is over, face-to-face (F2F) learning and traditional teaching methods will return to many courses (Cheng et al., 2021). However, studies have suggested that the use of digital learning elements was increasing and even common prior to the pandemic (Eickelmann & Vennemann, 2017; Zhang, 2020). Often the decision to include digital elements in teaching is up to the lecturers, and the degree to which digital learning is used varies widely (Howell & O’Donnell, 2017). Although there are many ways to use digital teaching, ultimately it is up to lecturers and their intention in using digital opportunities for teaching.

The implementation of digital elements can reduce teaching workload in the long term. The intention is related, among other things, to whether lecturers are advised to take on additional workloads to provide digital teaching (Handke, 2020). Other aspects, such as teaching commitments, may also influence the intention to use digital elements (Müller et al., 2018). Switching to digital teaching also involves a change in behavior for lecturers, which can be difficult and decrease positive intentions (Hargreaves, 2011). To motivate lecturers to use digital learning elements and thus improve teaching quality, it is necessary to understand the intention behind the use of digital learning tools and as well as the aspects that encourage lecturers to invest their time in digital teaching.

Previous research has found that both the intention to use digital learning elements and the actual use have increased (De Grove et al., 2012; Rafiq & Ameen, 2012). In addition, studies have been conducted on intention from the perspective of students (Shukla, 2021). From the lecturer’s perspective, usage depends on teaching experience, curriculum relatedness, academic field, and the time provided by the institution, among other things (Buzzard et al., 2011; De Grove et al., 2012; Zhang, 2020). The use of digital learning elements is mostly perceived positively, with students often preferring the use of digital elements more than their lecturers (Bond et al., 2018). Despite numerous studies, there is a knowledge gap in considering the workload required for lecturers to prepare and use digital learning elements as well as which factors encourage lecturers to use digital teaching. Although intention and use have been studied individually, they have not been combined to examine the intention–behavior relationship. Accordingly, the present study addresses this research gap.

The scope of the study, therefore, includes the workload that arises from digital teaching and the extent to which lecturers intend to integrate digital teaching elements into their teaching. In light of this, the following research questions will be address:

- RQ1: How are intentions formed regarding the use of digital learning elements?
- RQ2: Does intentional behavior also lead to actual behavior regarding the use of digital learning elements?

We used the reasoned action approach (RAA) to answer our research questions, which states that attitudes toward behavior, perceived norms, and perceived behavioral control determine individuals’ intentions and predict their behavior (Fishbein & Ajzen, 2011). This approach allows us to consider behavioral, social, and control aspects in decision-making about the intention and use of digital learning elements. We use a theory-based structural equation model to identify relevant reasons for usage decisions among the beliefs that lead to attitudes, perceived norms, and behavioral control. To the best of our knowledge, we are the first to apply RAA to the usage behavior of digital elements in teaching.

The main novelty of the study is that it focused specifically on education in higher education institutions. The consideration of digital tools and digital teaching methods refer to methods of higher education teaching. By exclusively interviewing university teachers, the structures and the teaching freedom of universities are also taken into account. This makes it particularly interesting for readers who are interested in the relationship between information and communication technology and education in higher education institutions. For both teachers and people in leadership roles, the results of this work offer recommendations for practice.

The remainder of this paper is organized as follows: The theoretical background section presents the particular characteristics of higher education teaching as well as approaches, methods, and elements of digital teaching. Furthermore, the results of previous studies are outlined, the research model according to the RAA is established, and hypotheses are drawn. Following this, the quantitative survey is presented as the chosen method, the sample is analyzed, and statistical tests are presented. The subsequent section presents the descriptive results and answers the research hypotheses. Thereafter, the study concludes by deriving the theoretical and practical implications of the study along with limitations and directions for future research.

## Theoretical background

### Higher education

According to the International Standard Classification of Education, education consists of different stages (UNESCO, 2012). In this context, university education awards a bachelor’s, master’s, or doctoral degree (Schneider, 2013). According to various other sources, university education is also referred to as higher education (Barnett, 1990; Caner, 2012; Regmi, 2012; Sharipov, 2020). Regarding the practice of university education, the main focus is on the practical implementation of knowledge transfer (Papadopoulos, 1998). Depending on the education level, different didactics should effectively impart knowledge (Kember, 2001). Another characteristic of higher education is that teachers have freedom in their teaching; in particular, they have control over the amount of teaching but not over what is taught or the methods used. This means that the use of digital teaching elements cannot be prescribed. Therefore, it is helpful to reflect university teaching separately from other educational stages. University faculties consist of various disciplines, and thus different course types are offered. Across disciplines, common course types include lectures, seminars, and exercise lessons (Smeby, 1996). Other typical types of university courses are colloquia, repetition courses, and projects (Reinmann et al., 2020) as well as school practical studies, project internships (Elster, 2002), and internships (Scicluna et al., 2014).

### Digital teaching

Before the advent of technology, teaching and learning practices were based primarily on F2F instruction, usually in a lecturer-led environment with person-to-person interaction (Caner, 2012). Digital teaching, in general, is characterized by the use of information and communication technologies (Gerasimova et al., 2018; Kruty et al., 2019; Kumar et al., 2020). Although digital teaching and digital learning are two different aspects, they both describe students’ digital education (Aretio, 2020), and, therefore, the term digital teaching elements can be used interchangeably with digital learning elements.

Digital learning approaches can be integrated into F2F teaching by incorporating digital elements into F2F courses. If the knowledge is imparted partly digitally and partly via the traditional F2F approach, this is referred to as blended learning (Bernard et al., 2014; Pacheco-Pereira et al., 2020) or hybrid learning (Ryan et al., 2016). Pure e-learning does not use F2F components, is delivered exclusively via digital learning elements, and can enable learning independent of time and place (Giugliano et al., 2020).

Digital learning approaches are implemented through various learning elements. Digital learning elements, therefore, represent the implementation or the tool through which knowledge is digitally delivered. Instructional elements can be grouped in a variety of ways, one of which is by how the learning element is perceived. The perception of learning elements can be classified into the three basic types of learning styles: auditory, visual, and kinesthetic (Schuemie et al., 2001). The kinesthetic learning style refers to active physical interaction with physical objects (Gilakjani & Ahmadi, 2011). In a digital environment, physical objects are often replaced by digital elements (Redström & Wiltse, 2018), and, for our purposes, we consider this to constitute “active” learning elements whereby learning takes place via interaction, similar to the kinesthetic learning approach. Learning styles can overlap or can be used in combination. In addition, they may occur individually or interactively (Gilbert & Han, 1999). Following Gilakjani and Ahmadi (2011); Redström and Wiltse (2018); Schuemie et al. (2001), exemplary learning elements, which are used in university teaching, can be assigned to different learning types:

- Visual: shared notes, Wikis, blogs, digital handouts, digital discussion forums, digital books, and digital models;
- Visual–interactive: synchronous conferences, meetings, or discussions;
- Visual–auditory: lecture videos, picture-in-picture videos, text-based videos, screencasts, fictional video, and infographic video;
- Auditory: podcasts and audio feedback;
- Auditory–interactive: audio interactions;
- Active: simulation, digital experiments, digital applications, augmented reality, virtual reality, process modeling, online quizzes, online surveys, online polls, video creation by students, joint editing of documents, digital whiteboard, electronic tests, online games, and E-portfolios.

The benefits of digital learning elements are individual and subject specific. The use of technology should always be viewed critically from the standpoint of educational usefulness (Castañeda & Selwyn, 2018). An effective blended learning environment is necessary to implement innovative pedagogically valuable approaches in higher education (Kintu et al., 2017). The blended learning approach can increase interaction with students (Kastner, 2020), reduce the actual teaching time (Förster et al., 2020), and allow learning to take place at an individual pace (Spadafora & Marini, 2018). In addition, the blended learning approach generates higher student satisfaction than pure F2F or online teaching (Sheikhaboumasoudi et al., 2018; Suwantarathip, 2019). Teaching methods with digital components, such as the flipped classroom concept and game-based learning, can also significantly increase learning performance (Chen & Tang, 2022; Nes et al., 2021). Other benefits include improved participation, attitude, motivation, pleasure, perceived learning, satisfaction, and practical skills, as well as increased learner competition (Nadi-Ravandi & Batooli, 2022). Positive effects can also be attributed to individual digital learning elements. For example, video lectures have been found to facilitate higher levels of cognitive activity than traditional lecture-based instruction (Shikino et al., 2021). The use of online discussion forums enhances student reflection (Saleh, 2020). In another study, a learning app was preferred by medical students over books, models, and atlases; the app was found to be the most helpful for the overall understanding of human anatomy (Rosario, 2021).

### Previous research

The intention from lecturers to use digital elements in the classroom has been investigated and confirmed several times, especially digital games (An, 2018; De Grove et al., 2012). A study in Pakistan showed that the actual use of digital elements in higher education is increasing as well as the demand for digitized content (Rafiq & Ameen, 2012). A majority (98%) of lecturers surveyed indicated that they are accustomed to using technology for many teaching purposes, such as giving presentations, preparing lessons, monitoring and assessing student progress, and creating reports (Joebgio & Akhyar, 2018). Digital learning elements are mainly communication tools, websites, office suites, and learning management systems such as interactive content, lecture-capture software, and multiplayer games (Buzzard et al., 2011). Previous studies mostly considered intention as a single variable without allowing further conclusions about different intention aspects such as behavioral, social, and control characteristics. One study examined the use of a digital tool in the area of coupon usage in shopping and confirmed a positive effect on usage intention with regard to all three aspects (Yakasai & Jusoh, 2015). In the area of digital teaching, however, these aspects have only been considered individually. It is, therefore, necessary to examine intention more closely using a theoretical model.

Previous research has already created a typology of lecturer attitudes toward information and communication technology (ICT) and examined the technology use of these clusters. Overall, 34.8% of ICT enthusiasts use ICT at least once a week for teaching. Partial ICT doubters use technologies at least once a week in 46% of the cases, whereas absolute doubters use them only in 32.3% of cases (Eickelmann & Vennemann, 2017). According to Eickelmann and Vennemann (2017), there is no correlation between attitude and actual usage. However, these results cast doubt on the classification of different lecturer types. Other aspects such as information literacy and digital skills have a moderating effect on general ICT use (Yu et al., 2017). For other personality traits, such as openness, agreeableness, and conscientiousness, a positive effect on behavioral intention to use ICT has been found, but, again, this was not specifically with respect to digital teaching (Dalvi-Esfahani et al., 2020). Findings indicate that lecturers who have better digital infrastructure in their faculty are more likely to use digital technologies (Soomro et al., 2020).

The extent to which digital competencies are acquired depends on the time made available by the institution for this purpose (Zhang, 2020). Studies have shown that the decision to use digital learning elements and integrate them successfully is primarily related to teaching experience and curriculum relatedness (De Grove et al., 2012). In addition, the academic field plays a role in the extent to which digital teaching is preferred. Research shows that lecturers and students in engineering, business, and education especially prefer technology in teaching. Comparatively, in life sciences and fine arts, technology is less preferred by lecturers in particular but also by students (Buzzard et al., 2011). In another study, it was found that, as long as instructors see a practice or type of knowledge as helpful in achieving their goals, they will engage in that practice or activity to acquire knowledge (Khong et al., 2022). In addition, the performance expectancy, effort expectancy, and social facilitation influence the intention of technology use in the classroom (Zhou et al., 2022). Another study indicated that facilitating conditions, subjective norms, and attitudes toward technology have a direct influence on prospective mathematics teachers’ intentions for technology use in their future classrooms; however, this study was not focused on teachers of higher education but, rather, on pre-service mathematics school teachers (Gurer, 2021).

With regards to technology use in examinations, research has found that, above all, the perceived usefulness influences the intention to use (Fink et al., 2022). However, the perceptions of the usefulness of various digital elements sometimes differ between students and instructors. One interesting difference is the percentage of lecturers (27%) who find lecture notes “not at all useful,” compared to 57% of students who find them “very useful” (Bond et al., 2018). The perceived usefulness of instant messaging, cloud storage, and learning management systems is also higher among students than lecturers. Overall, however, most of the elements are considered valuable rather than not helpful by both students and lecturers (Bond et al., 2018). Another study could also confirm the actual effectiveness of using digital educational resources. Self-organization components such as “situation analysis” and “goal setting” show higher values among students when digital elements are used (Drozdikova-Zaripova & Sabirova, 2020).

Few studies have addressed the relationship between intention and behavior. A study by Olugbara et al. (2020) found that the intention to use e-learning has a positive effect on actual integration. An older study by Almås and Krumsvik (2008) found a decreasing intention–behavior gap based on interviews and observations. However, this gap refers to the general use of ICT, which does not necessarily represent a learning element. Other studies examining this intention–behavior in general ICT use have found at least partial (Henderikx et al., 2017) or complete (Shah & Zhongjun, 2021) positive correlations.

Looking at regulations, many aspects of the legal and structural framework still need to be clarified. The impact of digital elements or online teaching on students’ study load has not yet been identified. Workload for content creation as well as preparation and post-processing times are not included in the specifications of teaching loads (Kleimann, 2008), nor has it been determined whether workload for online elements (e.g., in a chat room) can be credited to instructors, even if they are not physically present (Müller et al., 2018).

The literature is rich, but there are still many research gaps in theory and practice. Table 1 provides an overview of the topics covered by previous research. In addition to the content of the studies, whether there is a focus on (higher) education is also shown.

**Table 1:** Overview of previous research

Study specifics	Topic	Authors
Digital elements in higher education	Actual use	Rafiq and Ameen (2012)
Digital elements in general education	Intention	An (2018); De Grove et al. (2012)
	Attitude	Eickelmann and Vennemann (2017)
	Actual use	Buzzard et al. (2011)
	Intention–behavior relationship	Olugbara et al. (2020)
	Effects from external conditions on actual usage	De Grove et al. (2012)
Digital elements general	Intended use	Joebgio and Akhyar (2018)
	Intention–behavior relation	Yakasai and Jusoh (2015); Almås and Krumsvik (2008)
	Effects from personal traits on actual usage	Dalvi-Esfahani et al. (2020); Yu et al. (2017)
	Effects from external conditions on actual usage	Soomro et al. (2020); Zhang (2020)

In the context of higher education, only actual use has been examined, not intention or a possible intention–behavior gap. In the context of general education, studies have examined intent, actual use, and their relationship. However, these factors have only been examined individually, not combined. In addition, no theoretical approaches have been used to explain the use of different variables. Instead, the results of existing studies were determined based on a single variable. Furthermore, thus far, the learning elements used and effort required have been examined chiefly independently of one another. Many findings about the intention and use of digital elements in higher education can only be assumed on the basis of general results. Moreover, only a few learning element types have been investigated, not the entire range of possibilities. Ultimately, many studies are qualitative with only a few participants, and, therefore, they are only partially representative; a quantitative study with many participants is necessary. To provide a comprehensive view that is nevertheless specific to the topic, further investigation is required that considers different variables of use intention.

### Reasoned action approach and research model

In analyzing the intentions behind using digital learning elements, we use the RAA to consider the role of behavioral, social, and control aspects. The RAA is based on the widely accepted theory of reasoned action (Ajzen & Cote, 2008; Ajzen & Fishbein, 1973; Fishbein, 1967; Fishbein & Ajzen, 1977) and focuses on explaining individual behavior. It is also a fundamental model in the field of technology adoption in education (Songkram & Chootongchai, 2022). According to RAA, individual behavior is based on behavioral intentions, which are influenced by the following: (a) an individual’s attitude toward the behavior, represented by the belief the individual has regarding the behavior; (b) perceived norms, represented by the normative beliefs; and (c) the perceived behavioral control, represented by the possibility that the individual can control the behavior (Fishbein & Ajzen, 2011). By applying RAA to the context of digital learning elements, the theory provides several explanations for why lecturers intend to use digital learning elements as well as the extent of such use. We deliberately chose RAA because, unlike the Unified theory of acceptance and use of technology 2 (UTAUT2) or other technology acceptance models, it does not refer to a specific system. The RAA allows us to consider personal beliefs and thus also the didactic concept that goes hand in hand with digital learning elements. The focus should not be on the acceptance of a digital learning element but, rather, on the personal beliefs regarding its use.

First, behavioral beliefs capture a person’s values regarding digital learning elements, including aspects such as the adequacy of the information provided by the tool, the reliability of the tool, the ease of use of the tool, and the support provided by the tool to achieve excellent work results (Brailsford et al., 2013; Park et al., 2010). These beliefs lead to positive or negative attitudes toward the specific digital learning element. Positive feelings, in our case, refer to whether lecturers perceive the learning element as easy to use, are satisfied with the application, and are satisfied with the new digital learning element. It is expected that a person who has a more positive attitude will have a higher intention to use digital learning elements (Fishbein & Ajzen, 2011; Sheppard et al., 1988). This leads us to two hypotheses:

- **Hypothesis 1**: The stronger the behavioral beliefs regarding digital learning elements, the more positive an individual’s attitude regarding the elements.
- **Hypothesis 2**: The more positive the attitude of an individual regarding digital learning elements, the stronger the intention to use these elements.

Second, normative beliefs refer to the opinions of relevant people in the professional context concerning digital learning elements. Colleagues in the immediate work environment or in similar roles as well as supervisors are relevant referents in this regard (Fishbein & Ajzen, 1977). Students’ attitudes also influence the willingness of lecturers to use digital elements (Kreijns et al., 2013), so they are also included in this group of reference people. Normative beliefs lead to perceived norms, which are the perceptions of important reference persons regarding digital learning elements. Perceived norms can be expressed as the total of individual perceptual beliefs for all relevant referents (Ajzen & Fishbein, 1973; Fishbein, 1967; Fishbein & Ajzen, 1977). The normative influence concerning the use of digital learning elements is associated with lecturers seeking to exchange with others to gain experience about learning methods/learning elements and to exchange opinions (Trust et al., 2016). The next set of hypotheses reflects this:

- **Hypothesis 3**: The stronger the normative beliefs regarding digital learning elements, the more positive the perceived norms of an individual regarding the elements.
- **Hypothesis 4**: The more positive the perceived norms of an individual regarding digital learning elements, the stronger the intention to use these elements.

Third, control beliefs include factors that facilitate or impede the use of digital learning elements. Examples of conceptual components include ease of access to tools, lack of alternatives, costs and benefits of use, perceived levels of control in using the tool, and the respective importance of these digital learning elements. Control beliefs influence the perceived behavioral control, which refers to the individual’s assessment of whether they can use or have control over a specific digital learning element. Academic freedom is the self-control that invites lecturers to research, discover, publish, and teach as they believe is appropriate, without any authority of the rational methods (Osman, 2013), thus impacting the ability to conduct courses. Hence, we can derive the next set of hypotheses:

- **Hypothesis 5**: The stronger the control beliefs regarding digital learning elements, the more positive the perceived behavioral control of an individual regarding the elements.
- **Hypothesis 6**: The more positive the perceived behavioral control of an individual regarding digital learning elements, the stronger the intention to use these elements.

In summary, the more positive the attitude toward a particular behavior, the more positive the perceived norms, and the more positive the perceived behavioral control, the more likely the person is to use a digital learning element (Fishbein & Ajzen, 2011; Sheppard et al., 1988). Finally, our analysis focuses on actual behavior. We use the time of weekly usage effort of digital learning elements as an indicator to represent actual use. Based on the results from previous literature and the assumption that intentions do not always lead to the same behavior, generally there is a positive relationship between intention and behavior (Fishbein & Ajzen, 2011). Hence, we draw the following hypothesis:

- **Hypothesis 7**: The stronger the intention to use digital learning elements, the higher the actual use of these elements.

According to the RAA model, the actual control over performance has a direct influence on perceived behavioral control and moderates the relationship between intention and use (Fishbein & Ajzen, 2011). As a component of the actual control, the moderation effect has already been empirically confirmed for personal skills, at least with regards to general ICT use (Yu et al., 2017). Therefore, we hypothesize that this is also the case for the digital learning elements:

- **Hypothesis 8**: Personal skills regarding digital learning elements have a positive effect on the perceived behavior control.
- **Hypothesis 9**: Personal skills regarding digital learning elements positively moderate the intention–use relationship.

In addition to the RAA model, we include personal openness as an important personality trait in the context of digital teaching, which influences behavior in different situations (Bouchard Jr, 1994). Personal openness regarding ICT influences intention in this area (Dalvi-Esfahani et al., 2020) and also has a positive effect on the students choice of learning (Halder et al., 2010; Men & Noordin, 2019). We assume that personal openness to digital teaching has a positive effect on different model variables, and, in this respect, we state the last hypothesis:

- **Hypothesis 10**: Personal openness regarding digital teaching has a positive effect on a) attitude, b) perceived norms, c) perceived behavioral control, d) intention, e) personal skills, and f) use of digital learning elements.

**Fig. 1:**
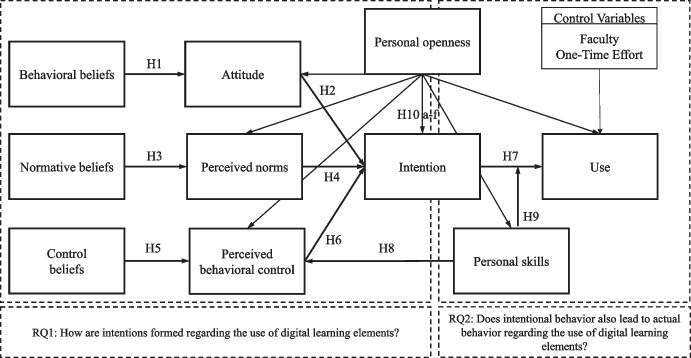
Research model, questions, and hypotheses

In addition to the RAA variables, we examine one-time effort in becoming familiar with digital learning elements and faculty affiliation as control variables. Figure 1 summarizes the research model, including the research questions and hypotheses.

## Research method

### Questionnaire

We used a questionnaire based on the RAA to collect data (Appendix Table 3). In the adaptation, we have closely followed the original framework and idea of Fishbein and Ajzen (2011). First, respondents were asked to indicate which digital learning elements they know and use and what they consider to be the most important digital learning element. In addition, the respondents selected the percentage of learning elements that can be reused in subsequent semesters and estimated the required one-time training effort and weekly effort to become familiar with them. Then they indicated the time required for available preparation and follow-up for the different course types they teach and the percentage of digital elements for those effort times. The questions thereafter referred to RAA-based beliefs using the perceived most important learning element as a reference object, and these were developed following Fishbein and Ajzen (2011), whose model provided the causal foundation of the questionnaire. However, we adapted their model to fit our investigation, as there is no universal RAA questionnaire that can be used for all contexts (Fishbein & Ajzen, 2011). Therefore, we based our questions on their existing framework but adapted the 7-point Likert scale items to the context of digital teaching in higher education. The main aspects of behavioral beliefs are ease of use and good delivery of instructional content. We related the normative beliefs to the reference groups of colleagues who taught and students who learned course content. Control beliefs were described by the ease of implementing digital learning elements and ease of access to them. We chose not to include the identification items from Fishbein and Ajzen’s (2011) sample questionnaire because it targets individuals in a more private context. The three beliefs were measured by a scale and weighted according to their importance, following Fishbein and Ajzen (2011). We focused on items concerning benefit, satisfaction, importance, gratification, and pleasure regarding attitude. These items are evaluative items in the original questionnaire. Our perceived norms and behavioral control items closely follow the sample survey. Intention to use digital learning items and current usage behavior were measured by frequency. In addition, we collected the control variables of personal skills regarding digital elements, openness to digital learning elements, and faculty affiliation.

### Sample

We collected data from lecturers at a mid-sized Germany university by sending the online questionnaire link to all teaching-related personnel. The university is an interesting example for investigating digital learning elements. Due to various projects regarding the digitalization of teaching at the university, many lecturers are engaged with digital learning, as confirmed in previous interviews. Because there is no compulsion to use digital teaching, the university is a good example to measure the actual personal intention and use. The questionnaire was sent to 1,697 professors and scientific employees of the university, but it was not possible to distinguish between teaching and non-teaching employees. Because the questionnaire was only addressed to persons with a teaching assignment, the target group is small. Overall, 142 questionnaires were completed and could be used for data analysis; 23.2% of the respondents belonged to Faculty of Philosophy, 20.4% to the Faculty of Mathematics and Natural Sciences, 17.6% to the Faculty of Economics and Social Sciences, 12.7% to the Faculty of Medicine, 9.9% to the Agricultural and Environmental Sciences Faculty, 6.3% to the Faculty of Computer Science and Electrical Engineering, 5.6% to the Faculty of Mechanical Engineering and Naval Architecture, 21% to the Faculty of Law, and 1.4% to the Language Center. In accordance with the university’s data-protection regulations, demographic data such as age or gender were not collected.

### Statistical tests

For our analysis, we used the partial least squares (PLS) method. We selected PLS and structural equation modeling because this combination can work efficiently with a wide range of sample sizes and with increased model complexity while making few restrictive assumptions about the data (Hair et al., 2011). We performed bootstrapping with a one-tailed test, a significance level of 0.05, and the recommended number of 5,000 samples (Hair Jr et al., 2021; Henseler et al., 2009), implemented via SmartPLS 3.3.9. Before conducting the analysis, we performed statistical tests to assess the quality of the reflective and formative measurement model and the overall quality of the structural model. We followed the procedure for PLS model assessment proposed by Henseler et al. (2009).

First, we evaluated the reliability and validity of the following reflective variables from the model: attitude, perceived norms, perceived behavioral control, intention to use, personal openness, and personal skills. All variables included composite reliability, which is higher than the required value of 0.8, confirming internal consistency reliability. All indicators fulfilled the criterion regarding indicator reliability confirmed by sufficient outer loadings. Furthermore, the discriminant validity is confirmed with values exclusively below 1 for the heterotrait–monotrait criterion. Convergent validity was evaluated with average extracted variance, for which all values are above 0.5, indicating a high-quality model.

Second, we conducted statistical tests to evaluate the formative variables of the model: behavioral beliefs, normative beliefs, and control beliefs. At the construct level, the criterion used for nomological validity was the relationship between the formative index and other constructs in the path model. Although behavioral beliefs and normative beliefs had a strong and significant effect on the other constructs in the path model, control beliefs did not. However, as the variables and items are considered suitable by previous research and the other statistical tests, we kept the control beliefs in our model. Regarding content validity, the outer weights of some indicators were removed because the values were not significant. In addition, the variance inflation factor values were sufficiently low, ranging between 1.027 and 1.352.

Third, we assessed the quality of the structural model. In our model, the different values can be considered moderate; only personal skills achieved a rather weak explanation by the individual items but still within a usable range. Furthermore, we used the Stone–Geisser Q^2^ criterion to assess the model’s predictive relevance. As the Q^2^ values of all constructs were above 0, we can assume high-quality variables in this test, which indicates a high predictive power for the model (Geisser, 1974). In addition to the statistical tests recommended by Henseler et al. (2009), we performed PLSpredict to test and interpret the predictive power of the individual indicators of the mediation variable. The procedure was performed following Shmueli et al. (2019). Overall, 19 of the 20 Q^2^ prediction values corresponded to a value above 0 and were thus used for further evaluation. All of those 19 values stood up to scrutiny, suggesting high predictive power for the indicators.

As the survey responses were given simultaneously (Campbell & Fiske, 1959), common method bias may have been present. To minimize this bias upfront, we followed Kortmann’s (2015) procedure regarding anonymity, confidentiality, the placement of dependent and independent variables, and the use of different scale types. In addition, a post-hoc test was conducted to assess data bias due to the common method. Harman’s one-factor test shows that the total variance of the most prominent factor was only 29.28%. Therefore, we can conclude that common method bias is either non-existent or negligible.

## Results

### Descriptive results

The most used digital learning element is synchronous online conferences or meetings, used by 74.6% of the lecturers. Online surveys (64.8%), online polling (62.7%), and digital handouts (62%) are also used by a large percentage of instructors. Other frequently used learning elements are lecture videos (57%), digital whiteboards (52.1%), e-books (45.1%), and digital discussion forums (42.3%).

Looking at the different categories of learning elements, the most used are elements in the active category, i.e., elements where students must consciously complete or participate in tasks. They account for 45.7% of the quantitative use of digital learning elements. The second most used category is visual learning elements (28.0%), followed by visual–auditory learning elements (12.0%). Visual–interactive learning elements account for 9.3%, auditory learning elements for 3.0%, and auditory–interactive learning elements for only 2.0% of usage. Synchronous conferences and meetings were selected as the most important digital learning element by 45.8% of respondents; 11.3% favored lecture videos, 10.6% digital whiteboards, and 8.5% digital handouts.

The respondents estimated the usage effort of one-time familiarization time with the digital element most important for their teaching at an average of 4.5 h (*n* = 142, md = 5, sd = 3.4). On a weekly basis, it was estimated that about 1.7 h (*n* = 142, md = 1, sd = 1.9) of effort is required to work with the digital element. On average, 57% of the elaborations for digital learning elements can be reused (*n* = 142, md = 70, sd = 33) in subsequent semesters. Considering the preparation time across all course types, the estimated share of digital learning elements is 36.6%. The effort for digital learning elements in artistic individual/group lessons is particularly high, accounting for 83.3% (*n* = 3, md = 80, sd = 6) of the total effort. For the other course types, somewhat more balanced percentages were found—lectures: 37.2% (*n* = 75, md = 30, sd = 26), exercises: 39.7% (*n* = 76, md = 30, sd = 28), seminars: 33.3% (*n* = 90, md = 30, sd = 23), colloquiums: 26.8% (*n* = 22, md = 20, sd = 25), repetitoriums: 48.0% (*n* = 5, md = 40, sd = 26), internships: 32.9% (*n* = 31, md = 30, sd = 28), project internships 45.0% (*n* = 10, md = 45, sd = 28), school practical studies: 30.0% (*n* = 10, md = 30, sd = 26), projects: 42.8% (*n* = 18, md = 45, sd = 21), and other: 45.0% (n = 2, md = 45, sd = 21).

Considering the personal beliefs of respondents, 95.8% believe it is essential to convey learning material in the best possible way. Only 66.2% of respondents saw the digital learning elements they frequently use as helpful; 63.4% saw these learning elements as a simple way of transferring knowledge, and 66.2% had a positive attitude toward the digital learning elements they used; 82.6% of instructors felt that it is under their control whether or not to use digital learning elements, and 66.7% intend to continue using important digital learning elements in the future.

### Results regarding the research model

**Fig. 2:**
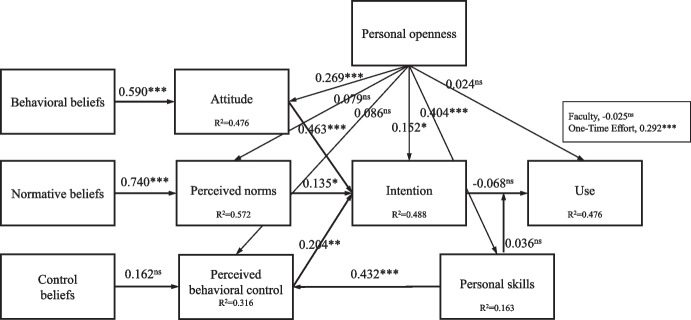
Results of the research model (* *p* < 0.05; ** *p* < 0.01; *** *p* < 0.001; one-tailed tests)

We calculated the underlying structural equation model to understand the intention to use digital learning elements and the impact by personal openness and skills in digital learning elements. Figure 2 shows the results.

We found empirical evidence for Hypothesis 1 (ß = 0.590***, f^2^ = 0.644) and Hypothesis 3 (ß = 0.740***, f^2^ = 1.252), showing that behavioral and normative beliefs are relevant antecedents. However, we did not find evidence corroborating Hypothesis 5 and control beliefs (ß = 162^ ns^, f^2^ = 0.031). Regarding Hypothesis 2 (ß = 0.463***, f^2^ = 0.306), Hypothesis 4 (ß = 0.135*, f^2^ = 0.033), and Hypothesis 6 (ß = 0.204**, f^2^ = 0.066), all three constructs were found to be predictors of intention to use. Therefore, attitude, perceived norms, and perceived behavioral control directly influence instructors’ intentions to use digital learning elements. It could not be empirically confirmed that intention to use is a predictor of actual use (Hypothesis 7: ß = -0.068^ ns^, f^2^ = 0.003). The lack of significant correlation here can thus be referred to as an intention–behavior gap. Hypothesis 8, that the non-behavioral variable of personal skills regarding digital learning elements influences the perceived behavior control, was confirmed (ß = 0.432***, f^2^ = 0.186). However, that personal skills moderate the relationship between intention and use could not be confirmed (Hypothesis 9: ß = 0.036^ ns^). Personal openness to digital learning elements had a significant influence on attitude (Hypothesis 10a: ß = 0.269***, f^2^ = 0.134), intention to use (Hypothesis 10d: ß = 0.152*, f^2^ = 0.037), and personal skills (Hypothesis 10e: ß = 0.404***, f^2^ = 0.195). There was no significant correlation for perceived norms (Hypothesis 10b: ß = 0.079^ ns^, f^2^ = 0.014^+^), perceived behavioral control (Hypothesis 10c: ß = 0.086^ ns^, f^2^ = 0.009), and actual use (Hypothesis 10f: ß = 0.024^ ns^, f^2^ = 0.000). Regarding control variables, a significant correlation was found for regular actual use (ß = 0.292**, f^2^ = 0.081) but not for faculty.

## Discussion

### Effort and usage of digital learning elements

Part of the motivation for this study was to find out what drives teachers to invest their time in engaging with and using digital instructional elements, and thus it is also essential to examine the effort that digital teaching requires. The most striking finding is the high standard deviation of some of the factors. In particular, one-time effort and reusability have a high standard deviation, possibly because these factors are affected by personal abilities or external influences. However, studies have shown that planning hours vary considerably between instructors independent of digital elements (Aydin, 2014; Merritt, 2016). Therefore, this result cannot necessarily be attributed to personal ability; it is probably more likely to be due to the general willingness of instructors. Müller et al. (2018) identified teaching commitment as an influencing factor in engaging with digital learning elements. In addition, the characteristics of digital learning elements, subject field, course type, and the time provided cannot be excluded as influencing variables, which is consistent with previous results (Buzzard et al., 2011).

All 142 respondents use at least one digital learning element for their courses; the highest number was 20 different digital elements, and the average was eight digital elements. The fact that digital elements are used by all or almost all teachers has already been identified by Joebgio and Akhyar (2018). In contrast with (Buzzard et al., 2011), communication tools were not considered the most important digital learning element, but learning elements for content delivery and course execution were. On the one hand, this may be because the demand for digital teaching is on the rise and an increasing number of digital options are available. On the other hand, this may be since the need for digital teaching continues to increase and more and more digital options are available (Rafiq & Ameen, 2012). However, external regulations are not to be neglected, which, due to the COVID-19 pandemic, have forced many lecturers to teach digitally.

The fact that learning elements in the active and visual categories account for the largest share (45.7% and 28%, respectively) should be considered with caution. They also contain the most elements, and the categorization does not consider the relevance or usage time. Many of these elements accompany the course and can also be used to prepare for, follow up on, or reinforce the actual course content. Incorporating digital components into the actual teaching is also reflected in the blended learning concept. Therefore, this result can be seen as an indicator that blended learning is becoming increasingly popular and widespread.

### Intention and actual use of digital learning elements

We used RAA to determine which beliefs lead lecturers to use digital learning elements and whether the intent to use matches actual use. This ensures a theoretically grounded consideration of the first research question and explains how the intention of using digital learning elements is formed.

Based on the structural equation model results, all three predictors have a significant and positive effect on the intention to use digital learning elements. These results are consistent with the general assumptions of Fishbein and Ajzen (2011), ICT-related research (Kreijns et al., 2013), and general educational research (Osman, 2013; Trust et al., 2016).

Looking at the variables in detail, the positive effect of behavioral beliefs on attitude toward digital learning elements was also confirmed by previous research findings. In addition, the influence of normative beliefs and perceived norms was confirmed based on the assumptions of the RAA model. A significant effect of control beliefs on perceived obsolescence control was not confirmed.

The RAA also allows us to answer the second research question and look more closely at the intention–behavior gap identified in the results. Although we expected a positive association between intention and use, this gap is not surprising. Some studies have identified a positive correlation between intention and use of e-learning (Olugbara et al., 2020), but a number of ICT-related studies have identified this gap either completely or at least partially (Almås & Krumsvik, 2008; Henderikx et al., 2017).

Many explanations can be given as to why usage behavior regarding digital learning elements ultimately does not lead to intended behavior. One is external influences, such as a missing crediting of the effort (Müller et al., 2018). Other reasons may involve the characteristics of the instructors. As personal skills often influence the actual use of ICT (Yu et al., 2017), we included them as a moderator variable in our model. Although personal skills as part of actual control also have a moderating influence on the intention–use relationship in the original RAA model (Fishbein & Ajzen, 2011), we did not find a significant effect. The strengthening effect on perceived behavioral control was confirmed according to the assumptions from the original model. In addition to strong personal skills as a variable of actual control, high scores were obtained regarding control beliefs and perceived behavior control. This suggests that actual control over digital learning elements is given and should not be a reason for the intention–behavior gap, even if it does not strengthen it.

A significant effect of personal openness was confirmed on intention but not on actual use. This is inconsistent with the general finding that different personality traits influence ICT use (Dalvi-Esfahani et al., 2020). The control variable of one-time expenditure was the only one to show a significant correlation with actual use. This suggests that a higher level of engagement with a digital learning element also leads to increased use in teaching.

Based on individual indicators of the survey, we assume the university’s actions and its employees and students could play a role in increasing actual use. The respondents have only a neutral opinion about whether the university provides enough information to use digital elements properly. More than half of the respondents stated that colleagues, students, and people in similar situations rarely recommend using digital learning elements. However, given personal control and personal capabilities concerning usage, we suggest that it is primarily other influences that affect the intention–behavior gap. Mandatory regulations or a lack of incentives, information, or time by the university or teachers may also influence the intention–behavior gap. Similar assumptions have been made and confirmed by Zhang (2020) and De Grove et al. (2012). Table 2 summarizes the influencing and non-influencing variables.

**Table 2:** Overview of influencing and non-influencing variables

	Intention	Usage
Variables with influence	Behavioral beliefs, normative beliefs, attitude, perceived norms, perceived behavioral control, personal openness, and personal skills	One-time effort regarding digital elements, personal abilities, general willingness, teaching commitment, subject field, course type, and provided time
Variables without influence	Control beliefs	Intention, personal openness, personal skills, and faculty

## Conclusion

### Theoretical contributions

In this study, we investigated the intentions and behaviors of university instructors concerning the use of digital learning elements. To determine the underlying intentions in detail, we adopted the RAA (Fishbein & Ajzen, 2011). We applied this approach to using digital learning elements for the first time. Our results empirically support the RAA especially regarding the influence of personal attitude, perceived norms, and perceived behavioral control on intended behavior. First, the relationship between intention and actual behavior could not be confirmed. As this also occurred frequently in previous studies and other fields of application, we included the variable personal skills and the personality trait of personal openness. Second, we could not find significant effects of the variables regarding the actual use or the moderation of the intention–use relationship. As personal openness to digital teaching significantly influences attitude, intention, and personal skills, the variable was nevertheless found to be relevant. Third, we recommend considering personal openness in future studies regarding intention and behavior, as the results provide evidence that it has more importance in terms of intention than presented in previous theoretical considerations. It is possible that other personality traits also have a significant impact. Therefore, in future research models, attention should also be given to those. Fourth, even if the assumptions made in the model are correct and important, other variables should be included in future studies, at least as control variables, to obtain a complete picture. This study has shown how important control variables can be for understanding results when initial theoretical assumptions are rejected.

### Practical contributions

Through digital learning elements, we hope to achieve the benefits of digital teaching. Only teachers of higher education were surveyed in the study, which is important for making appropriate recommendations. In accordance with previous research, we primarily considered the use of digital education in addition to traditional F2F instruction, where knowledge can be conveyed in the best possible way and learning outcomes can be optimized. Based on the findings, three key recommendations can be made:

1. Empowering the lecturer: To maximize the effectiveness of digital education, it is recommended that lecturers be provided with access to digital learning materials and the necessary training to effectively use them. This includes allocating sufficient time for lecturers to engage with digital learning elements and understand their functionality. For example, universities could offer workshops on the use of synchronous meetings, online polling, lecture videos, or digital whiteboards, or they could assign trained support staff to help with the preparation process.
2. Promoting intrinsic digital education: The use of digital education should not be compulsory but, rather, driven by the lecturer’s personal motivation. Universities should provide more information and resources to instructors, such as training sessions and peer-support groups, to increase their understanding and comfort with digital teaching tools. Additionally, regulations regarding workloads should be made more flexible, and instructors should be encouraged to allocate time to learning digital skills. For example, universities could offer incentives, such as continuing education credits, for lecturers who incorporate digital education into their curriculum.
3. Encouraging intention: Although the study did not find a significant relationship between intention and use, it is recommended that universities encourage instructors to adopt digital education by promoting personal openness and dismantling barriers. This could include creating accessible channels for instructors to share their experiences and best practices and providing opportunities for instructors to support each other. For instance, universities could organize forums or virtual workshops where instructors can discuss their experiences with digital education and receive feedback and guidance from their peers.

The research findings can also be used as a foundation for the development of educational programs and support services that aim to enhance teachers’ understanding and utilization of digital learning tools. These programs can include the following: workshops and training sessions that demonstrate the various digital learning tools and how they can be effectively incorporated into lesson plans; assistance and guidance for lecturers in overcoming obstacles and dispelling misperceptions about the usage of digital learning tools; personal-growth opportunities for educators that allow them to understand the factors that impact their motivation to use digital learning tools; online courses and webinars that provide lecturers with a deeper understanding of digital learning tools and their usage in the classroom; and mentorship programs that connect lecturers with experienced educators who have already successfully integrated digital learning tools into their teaching practice.

### Limitations and future research

As with any study, this study has some limitations that should be considered when looking at the results. Our sample refers to teaching staff at a medium-sized state-run university in Germany. Different results may be found in smaller, more prominent, or private universities. In addition, other effects are possible due to educational systems in other countries, even within Europe. Looking at the survey, it should be noted that intention and use were asked simultaneously. Although an attempt was made to avoid influencing the results using targeted wording, this cannot be completely ruled out.

Although we analyzed intention to use based on various predictors, actual use was only included in the model as a single-indicator variable. Other indicators could be taken into account and thus influence the results. Future research should include use as a latent rather than a manifest variable in the model, as this could help us understand the intention–behavior gap more precisely. Otherwise, the main focus should be researching why this gap exists and how it can be decreased. For this, external influences such as incentives or barriers could be examined more closely.

Furthermore, other personality traits should also be considered to obtain a holistic picture in addition to personal openness. To make more precise recommendations, the factors that influence personal openness in digital learning elements should also be examined. Among other things, digital fear can be seen as an inhibiting factor. It is also essential to compare and analyze the efforts of the individual learning elements in detail. In this study, the most important learning elements were considered in their entirety and personally. The voluntary effort required to implement and become familiar with digital learning elements to improve teaching should also be considered Finally, a detailed comparison between digital and analog teaching is required to compare the time spent on both.

## Data Availability

Data available on request from the authors.

## Appendix

Table 3

**Table 3:** Questionnaire

**1- Use of digital learning elements**: (1) Shared Notes (2) Wikis (3) Blogs (4) Digital handouts (5) Digital discussion forums (6) E-books Lecture videos (7) Digital models (8) Synchronous conferences, meetings or discussions (9) Lecture videos (10) Picture-in-picture videos (11) Text-based videos (12) Screencasts (13) Fictional video Infographic video (14) Podcasts (15) Audio feedback (16) Audio interactions (17) Simulations (18) Digital experiments (19) Digital applications (20) Augmented reality (AR) (21) Virtual reality (VR) (22) Process modeling (23) Online quizzes (24) Online surveys (25) Online voting (26) Video creation by students (27) Collaborative editing of documents (28) Digital whiteboard (29) Electronic tests (30) Online Games (31) E-portfolios (32) None (33) Other
**2- Most important digital learning element:** Please select what you consider to be the most important digital learning element for the delivery of your courses. (1) to (33)
**3- Reuse:** Please estimate the percentage of elaboration of (selection from 2) that you could reuse in subsequent semesters. (0%—100%)
**4- One-time training time:** Please estimate your effort of one-time training time to learn how to use (selection from 2) and to be able to use the element for your teaching. (0 h- 10 h and more)
**5- Weekly usage (Use):** Please estimate the weekly time you use (selection from 2) in your courses. (0 h- 10 h and more)
**6- Course types**: Please indicate through which course types you teach. (1) Lecture (2) Exercise (3) Seminar (4) Colloquium (5) Repetitorium (6) Artistic individual/group lessons (7) Internships (8) Project internships (9) School practical studies (10) Projects (11) Other
**7- Preparation and follow-up effort:** For (selections from 6), please indicate the weekly preparation and wrap-up time (in minutes) they require and the percentage of time spent using digital tools
**8- Behavioral beliefs** (BB1) (selection from 2) helps me to convey learning material in the best possible way. (BB2) It is very important to me to convey learning material in the best possible way. (7-Point-Likert-Scale: (1) do not agree at all—(7) completely agree)
**9- Attitude** (A1) The use of (selection from 2) is beneficial. (A2) The use of (selection from 2) is satisfactory. (A3) The use of (selection from 2) is important. (A4) The use of (selection from 2) is enjoyable. (5) The use of (selection from 2) pleases me. (7-Point-Likert-Scale)
**10- Normative beliefs** (NB1) Colleagues advise me to use (selection from 2). (NB2) I generally take advice from my colleagues very seriously. (7-Point-Likert-Scale)
**11- Perceived norm** (PN1) People I am influenced by advise me to use (selection from 2) in my courses. (PN2) People I care about advise me to use (selection from 2) in my courses. (PN3) People whose opinions I value advise me to use (selection from 2) in my courses. (PN4) People in a comparable situation to myself advise me to use (selection from 2) in my courses. (7-Point-Likert-Scale)
**12- Control beliefs** (CB1) I use (selection from 2) because they are easy for me to implement. (CB2) The simple implementation of (selection from 2) is very important to me. (7-Point-Likert-Scale)
**13- Perceived behavior control** (PBC1) It is under my control to use (selection from 2) agree agree fully. (PBC2) It is mainly up to me to use (selection from 2). (PBC3) I am convinced that I can use (selection from 2). (PBC4) If I really want to, I can use (selection from 3). (7-Point-Likert-Scale)
**14- Intention to Use** (I1) I will definitely use (selection from 2) in my next agree fully course. (I2) I intend to use (selection from 2) in my next course. (I3) I might use (selection from 2) in my next course. (7-Point-Likert-Scale)
**15- Personal skills** (PS1) The university currently provides exactly the information I need to use (selection from 2). (PS2) I always find it easy to use (selection from 2). (PS3) I find (selection from 2) to be a useful element for teaching content. (PS4) I do not need any help with the use and integration of (selection from 2) in courses. (7-Point-Likert-Scale)
**16- Personal openness** (PO1) When I hear about digital teaching, I look forward to experimenting with it. (PO2) Among my colleagues, I am usually the first to try digital teaching options. (PO3) I am generally reluctant to try out digital teaching options. (PO4) I like to experiment with digital teaching options. (7-Point-Likert-Scale)
